# Effect of lactoferrin on ram sperm motility after cryopreservation

**DOI:** 10.5713/ab.21.0561

**Published:** 2022-03-03

**Authors:** Jie Su, Caiyun Wang, Yongli Song, Yanyan Yang, Guifang Cao

**Affiliations:** 1 Inner Mongolia Key Laboratory of Basic Veterinary Science, Inner Mongolia Agriculture University, Hohhot 010018, China; 2 Department of Psychosomatic Medicine, Inner Mongolia Medical University, Huhhot 010030, China; 3 Research Center for Animal Genetic Resources of Mongolia Plateau, Inner Mongolia University, Huhhot 010021, China; 4 Inner Mongolia Academy of Agricultured and Animal Husbandry Sciences, Huhhot 010000, China

**Keywords:** Lactoferrin, Proteome, Ram, Sperm Cryopreservation

## Abstract

**Objective:**

The objective of this study was to analyse the differentially abundant proteins caused by freeze–thawing of ram sperm and explore candidate proteins of interest for their ability to improve ram sperm cryopreservation outcomes *in vitro*.

**Methods:**

Sperm were from three mature Dorper. Fresh and frozen sperm proteins were extracted, and the differentially abundant proteins were analysed by mass spectrometry. Among these proteins, lactoferrin (LTF) was selected to be added before cryopreservation. Next, sperm samples were diluted in Tris extender, with the addition of 0, 10, 100, 500, and 1,000 μg/mL of LTF. After thawing, sperm quality was evaluated by motility, plasma membrane integrity, mitochondrial activity and reactive oxygen species (ROS).

**Results:**

Cryopreservation significantly altered the abundance of 40 proteins; the abundance of 16 proteins was increased, while that of 24 proteins was decreased. Next, LTF was added to Tris extender applied to ram sperm. The results showed that sperm motility and plasma membrane integrity were significantly improved (p<0.05) by supplementation with 10 μg/mL LTF compared to those in the control group. There was no significant difference in mitochondrial activity between the 0 μg/mL group and other groups (p>0.05). Supplementation of the cryoprotective extender with 10 μg/mL LTF led to decreased ROS levels compared with those in the control and other groups (p<0.05).

**Conclusion:**

The LTF is an important protein during cryopreservation, and the addition of 10 μg/mL LTF to a cryoprotective extender can significantly improve the function of frozen ram sperm.

## INTRODUCTION

The study of frozen ram sperm began in 1950 when Emmens first cooled sheep semen to −79°C [1]. Jones found that the addition of a diluted solution of 1.5% dimethyl sulfoxide and 7% glycerine increased the motility of ram sperm [2]. Furthermore, Quinn et al [3] showed that freezing changes the chemical composition and ultrastructure of ram sperm. Visser and Salamon [4] used diluted Tris to freeze ram sperm and found the lambing rate to increase to up to 40%. When cryopreservation technology for ram sperm was developed in the 1990s, the conception rate still did not exceed 60%. Pool et al [5] injected melatonin *in vitro* to improve semen quality after freezing. A study by Keskin et al [6] showed that 5% glycol and 1.5% glycol+100 mM trehalose have a strong protective effect on the ultrastructural morphology of ram sperm. In recent years, researchers have improved the cryopreservation technology of ram sperm, but sperm quality has not clearly improved after cryopreservation.

Frozen sperm are widely used in the commercial cattle breeding industry; compared to that with frozen sperm in rams, the pregnancy rate with frozen cattle sperm is higher and more stable. Differences in sperm proteomes between ram and cattle have been confirmed. Zhu et al [7] used a data-independent acquisition (DIA)-mass spectrometry (MS) proteomic approach to identify 238 differentially abundant proteins in ram and bull sperm. A higher abundance of sperm proteins in rams is associated with immuno-protection and sperm capacitation, while proteins that inhibit sperm capacitation are more abundant in bulls [7]. Westfalewicz et al [8] compared the proteomes of fresh, equilibrated, and cryopreserved bull semen using 2-dimensional gel electrophoresis coupled with matrix-assisted laser desorption/ionization time-of-flight MS (MALDI-TOF MS). The results revealed 16 differentially abundant proteins whose abundance was significantly changed after cryopreservation. Most sperm proteins affected by equilibration and cryopreservation are membrane bound, and the loss of those proteins may reduce natural sperm coating [8]. He et al [9] identified 25 differentially abundant proteins associated with cryo-damaged ram sperm using 2-dimensional gel electrophoresis coupled with MALDI-TOF MS. The results demonstrated that mitochondrial disruption and oxidative stress may play important roles in the mechanism underlying damage due to the freeze–thaw process in ram sperm [9].

In this study, we found a significant changes in lactoferrin (LTF) after cryopreservation of ram sperm. The LTF is an iron-binding glycoprotein which is present in the seminal plasma of various mammals, such as horse, porcine, bovine and human [10–13]. The LTF was identified as a sperm-binding protien, which could play a biological role in sperm maturation and for protection or regulation of sperm in corpus and cauda [12,14]. Earlier studies have shown that addition of LTF in sperm extender may be useful for the characteristics of sperm after freezing and thawing [15–17]. Here, MS revealed a large degree of difference after freezing in Ram spern, and identified a variety of different proteins. Interestingly, we observed that expression level of LIF was significantly decreased in sperm after freezing.

## MATERIALS AND METHODS

### Animal care

The Institutional Animal Care and Use Committee of Inner Mongolia Agricultural University approved the experimental protocol employed in this study.

### Reagents

All reagents were purchased from Sigma–Aldrich Co. (St. Louis, MO, USA). unless indicated otherwise.

### Sperm collection and freeze–thaw process

Sperm (total 6,2/ram) were collected from three mature Dorper rams with an artificial vagina. Ejaculate motility was examined using a microscope (Eclipse 80i; Nikon Corporation, Tokyo, Japan). Sperm concentration was determined with a sperm density metre (Accucell; IMV Technologies, L’Aigle, France). The ejaculates were evaluated and accepted for evaluation if the following criteria were met: individual motility: >60% and total abnormality: <10%. Finally, three sperm samples was selected from three rams for MS. Sperm samples was diluted with Tris-A (291.7 mM Tris base, 95.1 mM citric acid, 70.2 mM fructose, 20% egg yolk, 19 mg/mL tylosin, 50 mg/mL lincomycin, 100 mg/mL azithromycin, 99.01 mg/mL gentamicin, pH 6.8) to 4.0×10^8^ sperm/mL. Diluted samples were slowly cooled to 4°C and allowed to equilibrate for 90 min. After equilibration, equal amounts of Tris-B (291.7 mM Tris base, 95.1 mM citric acid, 70.2 mM fructose, 20% egg yolk, 12% glycerine, 19 mg/mL tylosin, 50 mg/mL lincomycin, 100 mg/mL azithromycin, 99.01 mg/mL gentamicin, pH 6.8) were added to sperm samples at 15-min intervals. The final concentration of the frozen sperm was 2.0×10^8^ sperm/mL. Samples of sperm were frozen in straws using a computer-controlled automatic freezer. After at least two days in liquid nitrogen, the samples were thawed at 37°C for 1 min. Thawed sperm were transferred to centrifuge tubes and held in a water bath at 37°C during the entire experimental process.

### Extraction of sperm proteins

The urea/thiourea method was used to extract sperm proteins [8]. The fresh and frozen sperm samples were centrifuged at 10,000×g at 4°C for 60 min. The sperm pellets were washed three times with phosphate-buffered saline (PBS) and centrifuged at 900×g for 5 min at 4°C. The sperm pellets were dissolved in lysis buffer (7 mol/L urea, 2 mol/L thiourea, 4% (w/v) 3-[(3-cholamidopropyl)-dimethylammonio]-1-propanesulfonate, 2% (w/v) dithiothreitol, 1% (v/w) protease inhibitor cocktail). Protein concentration was measured by the Bio-Rad Bradford protein assay using bovine serum albumin (BSA) as a standard.

### Mass spectrometry

The MS and DIA experiments were performed. Raw data were acquired on a Q Exactive HF mass spectrometer coupled with an Easy nanoLC 1000 chromatograph (Thermo Scientific, Waltham, MA, USA). Briefly, 3 μg of peptides was loaded onto a home-packed column (150 m×15 cm, ReproSil-Pur C18-AQ, 1.9 μm, Dr. Maisch GmbH, Ammerbuch, Germany) with an integrated spray tip. The peptides were separated with a 180-min gradient (from 5% to 13% B in 13 min, from 13% to 30% B in 145 min, from 30% to 45% B in 15 min, from 45% to 95% B in 1 min, and held at 95% B for 6 min; buffer A was 0.1% (v/v) formic acid in water, and buffer B was 0.1% (v/v) formic acid in ACN) at a flow rate of 600 nL/min. The column was coupled to a column heater (Allwegene Technology Co., Ltd. Beijing, China), and the temperature was set to 60°C. The sample raw data obtained by DIA were imported for quantitation, all detected peaks were reintegrated by using the mProphet peak-scoring model (each dataset and iteration were trained independently), and the q-value annotation was added to each peak. Then, the protein quantitation results were exported with MSstats [18] for further bioinformatics analysis.

### Western blotting

Differentially abundant proteins were screened, and we selected the LTF protein for western blot analysis to validate its abundance in fresh and frozen–thawed ram sperm.

Extracted sperm proteins were separated by sodium dodecyl sulfate-polyacrylamide gel electrophoresis using a 12% gel. After transfer onto a PVDF membrane by electroblotting, the membrane was incubated overnight with anti-LTF polyclonal antibody as the primary antibody at a 1:1,000 dilution at 4°C, washed in TBS-T, and incubated with goat anti-rabbit IgG H&L/HRP antibody (immunoglobulin G [IgG]; horseradish peroxidase [HRP]) (BS-0295G-HRP; Bioss, Beijing, China) at a 1:2,000 dilution for 2 h. After rinsing the membranes in TBST again, the protein bands were visualized using the Odyssey infrared imaging system (LI-COR Biosciences, Lincoln, NE, USA). The films were scanned, and all the data were entered into SPSS statistical software (SPSS, Inc., Chicago, IL, USA) and analysed using a two-sample t test.

### Immunofluorescence

Smears of the final suspension of sperm in diluent were air-dried on microscope slides at room temperature and fixed in methanol, after which the slides were washed three times in PBS for 10 min and blocked for 30 min at room temperature in 5% BSA. Sperm were incubated overnight at 4°C with anti-LTF (BS-5810R; Bioss, China) (1:400). For the negative control, PBS was used instead of primary antibody. Next, sperm were incubated with Alexa Fluor 555-conjugated goat anti-rabbit IgG H&L (1:400) (BS-0295G-AF555; Bioss, China) for 1 h at 37°C. Finally, sperm were incubated in the dark at 37°C for 10 min for staining with DAPI (4′,6-diamidino-2-phenylindole). Images were obtained using a confocal laser microscope (LSM; Zeiss, Oberkochen, Germany); red fluorescence on the images indicated positivity.

### Lactoferrin treatment of sperm

Each ejaculate was diluted with Tris extender containing LTF (10, 100, 500, and 1,000 μg/mL) or without LTF (control).

### Assessment of semen quality parameters

#### Sperm motility

Samples were adjusted to a concentration of 5.0×10^6^ sperm/mL, placed on a slide, covered with a coverslip and assessed by microscopic observation on a warm (38°C) stage. Motility is expressed as the percentage of motile sperm with moderate to vigorous linear progressive movement in all microscopic fields examined. At least 200 sperm were tracked and analysed at 38°C to assess the proportion of motile sperm.

#### Sperm membrane integrity

Sperm membrane integrity was assessed using a combination of 6-carboxyfluorescein diacetate (6-CFDA) and propidium iodide (PI) as previously described [19]. Take 50 μL of sperm and dilute with 150 μL of Tris without glycerol or egg yolk containing 5 μL of 6-CFDA (0.46 mg/mL) and 20 μL PI (0.5 mg/mL) and incubated in the dark at 37°C for 10 min, and 0.5% glutaraldehyde was then added for fixation. Sperm membrane integrity was observed with an Olympus FluoView (ver. 2.1a; Olympus, Tokyo, Japan). At least 200 sperm per slide were examined and scored for dye deposition. Sperm displaying green–red or red fluorescence were considered to have membrane damage, whereas those displaying green fluorescence were considered to have an intact membrane.

#### Sperm acrosome integrity

Sperm acrosome integrity was assessed using fluorescein isothiocyanate conjugated to peanut agglutinin (FITC-PNA) and by PI staining as described by Bucak et al [20]. Take 50 μL of sperm and mix with 10 μL of FITC-PNA (0.12 mg/mL) and 2.5 μL of PI. The samples were incubated at 37°C for 10 min in the dark, and 0.5% glutaraldehyde was then added for fixation. Sperm acrosome integrity was observed with an Olympus FluoView (ver. 2.1a; Olympus, Japan). At least 200 sperm per slide were examined to record the percentage of fluorescent, acrosome-intact sperm. Sperm displaying bright green or patchy green fluorescence were considered to have a non-intact or damaged acrosome, whereas cells that did not display green fluorescence in the acrosome cap were regarded as having an intact acrosome.

#### Sperm mitochondrial activity

Sperm mitochondrial activity was assessed with JC-1 with a protocol from Gravance et al [21]. Take 50 μL of sperm sample and dilute with 150 μL of Tris without glycerol or egg yolk containing 5 μL of the lipophilic cationic fluorochrome JC-1 (0.15 mM in DMSO) and incubated at 37°C for 10 min in the dark, and 0.5% glutaraldehyde was then added for fixation. At least 200 sperm per slide were examined to record the percentage of fluorescent sperm. Sperm were classified as having high mitochondrial membrane activity when the midpiece emitted yellow/orange fluorescence and low mitochondrial membrane activity when green fluorescence was emitted.

#### Intracellular reactive oxygen species measurements

Sperm intracellular reactive oxygen species (ROS) were assessed using 2′,7′-dichlorofluorescin diacetate (DCFH-DA). Take 50 μL sperm samples and dilute with 150 μL of Tris without glycerol or egg yolk containing 5 μM DCFH-DA and incubated at 37°C for 30 min in the dark, and 0.5% glutaraldehyde was then added for fixation. DCFH oxidation by ROS from plasma generated DCF, and DCF fluorescence intensity was read (with a SpectraMax reader) at an excitation wavelength of 485 nm and an emission wavelength of 530 nm.

### Statistical analysis

Values obtained upon assessment of the 5 sperm quality parameters (motility, membrane integrity, mitochondrial activity, Intracellular ROS measurements) are expressed as mean±SEM. The statistical software SPSS ver. 15.0 for Windows (SPSS Inc., USA) was used for the analysis. A probability level of <0.05 was considered to indicate significance.

Proteins with a false discovery rate below 1% were exported for statistical analysis. A t-test (α of 0.05) was used to compare fresh versus frozen sperm protein samples. High fold change of >1.5 or <0.5 was applied as an additional cut-off to ensure statistical significance, and p values were corrected for multiple testing by controlling for a 1% false discovery rate during analysis using the Benjamini–Hochberg method.

## RESULTS

### Significantly differentially abundant proteins after the cryopreservation of ram sperm

Sperm were screened with an abundance ratio ≥1.5 or ≤0.5 and a p≤0.05 as screening conditions, which identified 40 differentially abundant proteins when fresh sperm and frozen sperm were compared; among these proteins, 16 were upregulated in frozen sperm, and 24 were downregulated (Table 1).

**Figure 1:**
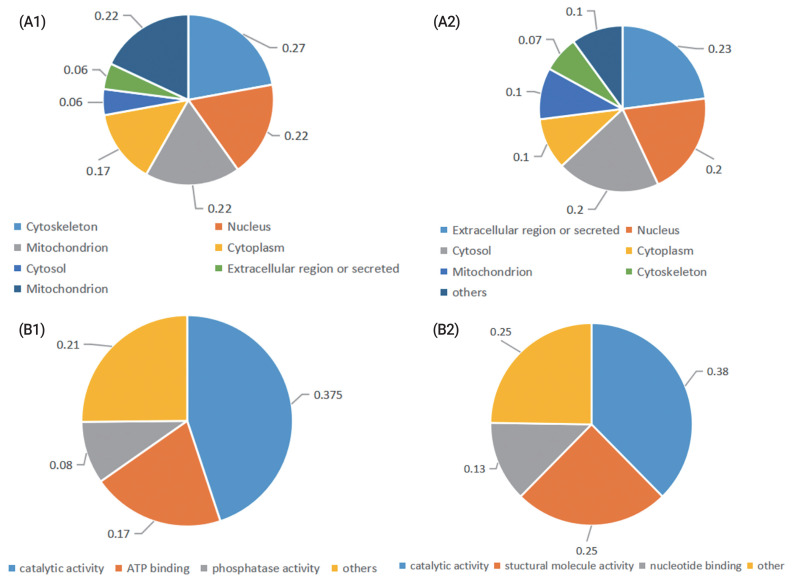
Classification of the identified proteins in fresh and cryopreserved ram sperm. According to their (A) subcelluar location, (B), molecular function. (A1), (B1) Proteins were upregulated in frozen sperm; (A2), (B2) Proteins were downregulated in frozen sperm.

Gene ontology (GO) annotation of enriched cellular components revealed that most proteins whose abundance was increased after cryopreservation are present in the cytoskeleton, nucleus, mitochondrion, or cytoplasm. The GO annotation of molecular function revealed that the most prevalent terms represented were catalytic activity, structural molecular activity and nucleotide binding. The differentially abundant proteins whose abundance decreased after cryopreservation were located in the extracellular region or secreted or found in the cytosol, nucleus. The GO annotation of molecular function revealed that the most prevalent represented terms were catalytic activity, ATP binding and phosphatase activity (Figure 1).

### Western blotting

**Figure 2:**
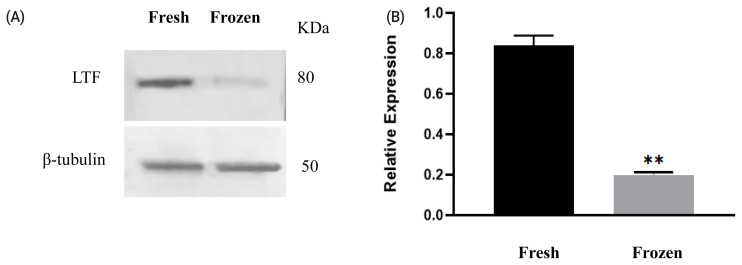
Expression of lactoferrin (LTF) before and after cryopreservation. Western blotting analysis of protein expression in fresh and freeze-thawed ram sperm (A). Relative expression levels of LTF in fresh and frozen–thawed sperm (B). Values represent the average± standard error of the mean from three gels per group. Asterisks indicate a statistically significant difference from the fresh group: ** p<0.001 from one-way analysis of variance with Duncan’s post hoc analysis.

Among the differentially abundant proteins, LTF was selected to validate the proteomics results. The changes in protein levels determined by western blot analysis (shown in Figure 2) were generally consistent with the variations recorded in the LC–MS/MS analysis. The LTF level was decreased in ram sperm after freezing.

### Immunofluorescence

**Figure 3:**
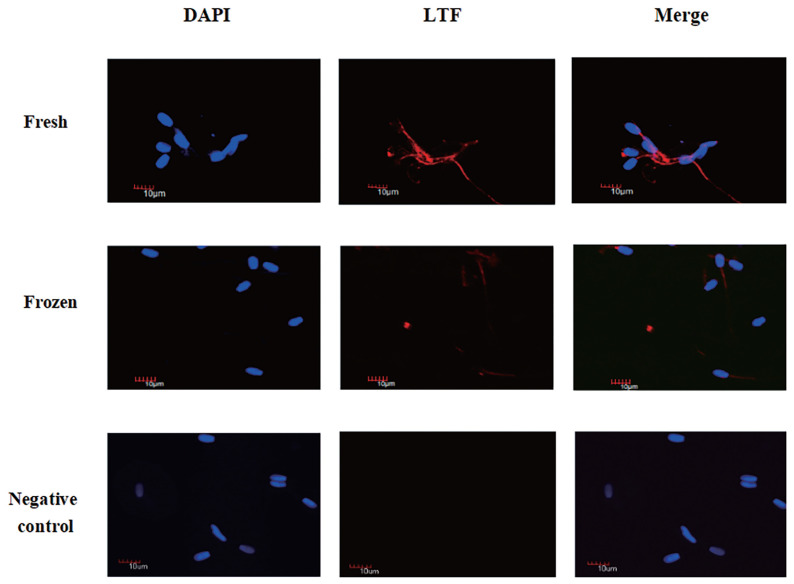
Immunofluorescence localization analysis of lactoferrin (LTF) in ram sperm. Immunofluorescence localization analysis of LTF in ram sperm. Staining was performed at 400×magnification. DAPI (4′,6-diamidino-2-phenylindole) (blue) and LTF (red).

Assessment of the LTF immunofluorescent signal showed that LTF (Figure 3) was clearly visible but minimally abundant in the semen flagellum. The abundance of LTF was decreased in frozen semen compared to fresh sperm.

### Lactoferrin treatment of frozen ram sperm

The effects of LTF supplementation (L9705; Sigma, USA) of the cryoprotective extender on ram sperm qualities are shown in Table 2. The motility and plasma membrane integrity were significantly improved (p<0.05) by supplementation with 10 μg/mL LTF. There was no significant difference in mitochondrial activity between the control group and the other groups. Supplementation of the cryoprotective extender with 10 μg/mL LTF led to decreased ROS levels compared with those in the control and other groups.

## DISCUSSION

In the present study, MALDI-TOF/TOF MS was used to determine changes in ram sperm proteins after cryopreservation. A total of 40 proteins underwent significant changes in abundance. Twenty-four sperm proteins decreased in abundance during cryopreservation, and 16 sperm proteins increased in abundance. The proteomes of ram sperm before and after freezing have been described using different proteomic technologies. Pini et al [22] reported that cryopreservation significantly altered the abundance of 51 proteins, among which the levels of 27 proteins increased, while those of 24 proteins decreased in frozen–thawed ram sperm. In another study, He et al [9] identified 21 proteins whose abundance differed between fresh and frozen–thawed ram sperm by two-dimensional electrophoresis. The abundance of 13 sperm proteins decreased, while that of 8 proteins increased during cryopreservation. Cryopreservation of sperm involves many separate processes, each of which may contribute to alteration of the sperm proteome. Few of the proteins that we identified as significantly altered in abundance following cryopreservation have been identified in other studies. This may be the result of differences in the cryoprotective extender or the methods used for quantitation of the differences. Most of the up-regulated proteins were involved in an apoptosis–stress response and several biological processes increased during sperm capacitation or cryo-capacitation; meanwhile, most of the down-regulated proteins were implicated in immunity and other relevant sperm functions. The cause of the increase in protein abundance upon cryopreservation is not known.

The loss of proteins itself may lead to sublethal sperm damage and lower sperm function after thawing. In our study, 24 sperm proteins decreased in abundance during cryopreservation. Seven of these proteins, DEFB133, LGALS3BP, LTF, BPIFA1, PGLYRP1, GLOD4, and GLB1, are located in the extracellular region or are secreted. Among these proteins, LTF was identified as a sperm-binding protein that may play a role in the protection and regulation of sperm activity [10, 12]. Kobayashi et al [15] reported that LTF may be useful for the modulation of bovine sperm viability, motility, capacitation state, and cryopreservation *in vitro*. Martins reported that LTF supplementation was beneficial in protecting stallion sperm during freezing, as it increased the percentage of sperm with a functional membrane and decreased lipid oxidant agents [17]. It is difficult to state the effect of the other proteins differentially expressed genes on sperm after freezing, because we could not find the related functional annotations in GenBank and related research. Therefore, we chose LTF for further research.

Cryopreservation has been reported to induce damage to sperm and the overproduction of ROS. Excessive generation of ROS during cryopreservation leads to major changes in proteins, lipids and carbohydrates in the sperm membrane, resulting in the loss of sperm motility [23]. Fe^3+^ is present at a very low concentration in almost all solutions and can catalyse the formation of hydroxyl radicals from O_2_ and H_2_O_2_. The presence of Fe^3+^, a catalytic transition metal, hastens the initiation of lipid peroxidation [24]. LTF can remove Fe^3+^ from the extracellular space and prevent its catalytic effects. The results in the present study revealed positive and negative effects of LTF at different concentrations. Supplementation of the cryoprotective extender with LTF at a concentration of 10 μg/mL resulted in a significant increase in sperm motility and plasma membrane integrity (p<0.05), while ROS levels were decreased compared with those in the control and other groups.

## CONCLUSION

The LTF decreased in abundance during cryopreservation. *In vitro* experiment suggested that the addition of 10 μg/mL LTF to a Tris extender can significantly improve the function of frozen ram sperm. LTF play an important role during cryopreservation of ram sperm and can significantly improve the function of frozen ram sperm.

## Figures and Tables

**Table 1:** Proteins differentially abundant between fresh ram sperm and frozen sperm

Uniprot access number	Protein name	Mean fresh	Mean frozen	p-value	Fold change (Frozen/fresh)
K4P1S5	Peptidyl-prolyl cis-trans isomerase	3,707,874,819	5,599,710,656	0.01341	1.51022106
W5PSZ6	Testis anion transporter 1	1,767,609,471	2,699,264,635	0.02516	1.52707070
W5PUU4	SPEM family member 2	1,274,951,062	2,023,261,858	0.03450	1.58693295
W5PUD8	Mitochondrial amidoxime reducing component 2	178,184,674	284,574,082	0.04709	1.59707384
A0A0A7AK60	Cytochrome b	764,492,041	1,228,232,142	0.00063	1.60659899
W5PUY0	RB binding protein 7	48,731,112	82,566,170	0.00375	1.69432150
W5Q5U5	Keratin 4	35,775,798,207	62,253,734,428	0.00319	1.74010749
W5Q665	Keratin 13	13,295,282,486	23,506,798,852	0.00086	1.76805561
W5Q611	Keratin 1	2,060,133,276	4,077,639,334	0.03506	1.97930851
W5QEU2	Poly [ADP-ribose] polymerase	1,248,508,039	3,097,205,302	0.00031	2.48072515
W5Q160	Keratin 10	1,514,687,200	4,218,898,678	0.00045	2.78532668
W5QFN4	Succinyl-CoA:3-ketoacid-coenzyme A transferase	732,737,023	2,376,841,286	0.00913	3.24378489
W5PLV3	RAB5B, member RAS oncogene family	32,016,601	110,367,103	0.00210	3.44718363
W5PX22	Small nuclear ribonucleoprotein Sm D3	197,008,375	56,008,424	0.00909	3.51747753
W5Q5S8	Keratin 79	229,298,108	827,888,775	0.00975	3.61053469
W5PAI8	Nicotinamide-nucleotide adenylyltransferase	9,930,950,124	2,368,220,178	0.00423	4.19342349
W5PVP5	Adenine phosphoribosyltransferase	2,285,213,650	91,102,607	0.04831	0.00880968
W5PST9	Beta-defensin	91,238,445,779	5,078,394,089	0.00015	0.05407310
A4GZY3	Seminal vesicle protein	82,867,236,507	5,838,425,974	0.01171	0.07699096
W5PMZ6	Alpha-mannosidase	14,644,733,570	1,934,251,271	0.00096	0.13207828
A0A075W0S2	BPI fold containing family A member 1	14,644,733,570	1,131,967,594	0.00257	0.16079215
W5QBA7	3-phosphoinositide dependent protein kinase 1	14,869,965,871	1,372,953,284	0.02388	0.20384992
W5NZ70	Galectin 3 binding protein	380,648,426	81,347,833	0.00735	0.21370857
W5P3C6	Beta-hexosaminidase	15,535,334,719	3,423,356,047	0.00175	0.22035933
W5PP77	Glyoxalase domain containing 4	359,971,895	50,193,314	0.03229	0.24004450
W5P7T9	KIAA1468	380,648,426	29,867,839	0.01483	0.26614296
D3G9G3	Lactoferrin	153,769,000,000	43,836,589,114	0.00057	0.28508034
W5PG95	Heat shock 70 kDa protein 1A/1B	15,535,334,719	2,557,982,351	0.02105	0.28715324
B5SZL5	Malic enzyme	189,727,532	54,480,876	0.00300	0.28910439
W5NTZ8	Reticulocalbin 2	1,769,988,417	295,039,268	0.04642	0.30642427
W5Q0T3	Lactoylglutathione lyase	11,350,485,617	2,719,761,011	0.02299	0.36115272
W5QC38	Tubulin alpha chain	153,769,244,331	27,080,922,435	0.03532	0.38446493
W5Q1M0	Beta-galactosidase	189,727,533	30,198,394	0.01315	0.40082802
W5NT19	Sperm associated antigen 1	10,568,960,720	1,843,614,094	0.03940	0.44507809
W5PYU5	Spalt like transcription factor 4	34,194,681	6,912,804	0.00517	0.45010647
B2LYK4	RAS oncogene family-like 4	40,454,561	18,208,859	0.00517	0.45010647
W5PCB3	Small VCP interacting protein	633,518,665	305,761,430	0.01191	0.47078067
W5P1Q0	AP complex subunit beta	622,622,997	575,674,784	0.03461	0.47234615
W5PSZ8	Triokinase and FMN cyclase	23,864,858,955	12,281,003,018	0.00675	0.47652161
C5IJA4	ATXN3	26,677,404,693	12,712,359,829	0.00675	0.47652161

1) Differentially expressed (>1.5-fold or ≤0.5) proteins of sperm after cryopreservation.

**Table 2:** Effects of lactoferrin on the motility, mebrane integrity, mitochondrial activity and ROS content in ram sperm berore and after cryopreservation

Items (%)	0 μg/mL	10 μg/mL	100 μg/mL	500 μg/mL	1,000 μg/mL
Motility	26.7±6.07^a^	41±3.2^b^	32.7±3.9^ab^	30.2±3.8^ab^	27.0±6.0^ab^
Membrane integrity	12.4±0.3^a^	17.9±0.1^b^	12.1±0.7^a^	11.7±0.4^a^	11.0±0.2^a^
Mitochondrial activity	24.2±6.1^a^	32.9±6.0^a^	24.7±3.2^a^	25.5±4.8^a^	21.5±3.5^a^
ROS	81.5±5.5^a^	61.5±1.5^b^	68.5±1.5^a^	74.2±3.2^a^	78.5±5.5^a^

Values represent averages±standard error of the mean (n = 3).

ROS, reactive oxygen species.

a,b Different superscript letters in the same row indicate significant differences (p<0.05).

## References

[1] Larsson K, Einarsson S (1976). Influence of boars on the relationship between fertility and post thawing sperm quality of deep frozen boar spermatozoa. Acta Vet Scand.

[2] Jones RC (1965). The use of dimethyl sulphoxide, glycerol, and reconstituted skim milk for the preservation of ram spermatozoa. II. The influence of diluent composition and processing time during freezing to minus 79 degrees C with dimethyl sulphoxide or glycerol or both compounds. Aust J Biol Sci.

[3] Quinn PJ, White IG, Cleland KW (1969). Chemical and ultrastructural changes in ram spermatozoa after washing, cold shock and freezing. J Reprod Fertil.

[4] Visser D, Salamon S (1973). Fertility of ram spermatozoa frozen in a tris-based diluent. Aust J Biol Sci.

[5] Pool KR, Rickard JP, Tumeth E, Graaf SD (2020). Treatment of rams with melatonin implants in the non-breeding season improves post-thaw sperm progressive motility and DNA integrity. Anim Reprod Sci.

[6] Keskin N, Erdogan C, Bucak MN, Ozturk AE, Dursun S (2020). Cryopreservation effects on ram sperm ultrastructure. Biopreserv Biobank.

[7] Zhu W, Cheng X, Ren C (2020). Proteomic characterization and comparison of ram (Ovis aries) and buck (Capra hircus) spermatozoa proteome using a data independent acquisition mass spectometry (DIA-MS) approach. Plos One.

[8] Westfalewicz B, Dietrich MA, Ciereszko A (2015). Impact of cryopreservation on bull (Bos taurus) semen proteome. J Anim Sci.

[9] He Y, Wang K, Zhao X, Zhang Y, Ma Y (2016). Differential proteome association study of freeze-thaw damage in ram sperm. Cryobiology.

[10] Inagaki M, Kikuchi M, Orino K, Ohnami Y, Watanabe K (2002). Purification and quantification of lactoferrin in equine seminal plasma. J Vet Med Sci.

[11] Pearl CA, Roser JF (2008). Expression of lactoferrin in the boar epididymis: effects of reduced estrogen. Domest Anim Endocrinol.

[12] Pearl CA, Roser JF (2014). Lactoferrin expression and secretion in the stallion epididymis. Reprod Biol.

[13] Zumoffen CM, Massa E, Caille AM, Munuce MJ, Ghersevich SA (2015). Effects of lactoferrin, a protein present in the female reproductive tract, on parameters of human sperm capacitation and gamete interaction. Andrology.

[14] Jin YZ, Bannai S, Dacheux F, Dacheux JL, Okamura N (1997). Direct evidence for the secretion of lactoferrin and its binding to sperm in the porcine epididymis. Mol Reprod Dev.

[15] Kobayashi J, Suda Y, Takada N (2006). 14 motility and fertility of bull spermatozoa frozen in egg yolk extender supplement with lactoferrin. Reprod Fertil Dev.

[16] Kobayashi J, Sasaki A, Watanabe A (2021). Effects of exogenous lactoferrin on characteristics and functions of bovine epididymal, ejaculated and frozen-thawed sperm. Anim Sci J.

[17] Martins HS, Da Silva GC, Cortes SF (2018). Lactoferrin increases sperm membrane functionality of frozen equine semen. Reprod Domest Anim.

[18] Choi M, Chang CY, Clough T (2014). MSstats: an R package for statistical analysis of quantitative mass spectrometry-based proteomic experiments. Bioinformatics.

[19] Silva ECB, Cajueiro JFP, Silva SV, Soares PC, Guerra MMP (2012). Effect of antioxidants resveratrol and quercetin on in vitro evaluation of frozen ram sperm. Theriogenology.

[20] Bucak MN, Ataman MB, Bapnar N (2015). Lycopene and resveratrol improve post-thaw bull sperm parameters: sperm motility, mitochondrial activity and DNA integrity. Andrologia.

[21] Gravance CG, Garner DL, Baumber J, Ball BA (2000). Assessment of equine sperm mitochondrial function using JC-1. Theriogenology.

[22] Pini T, Rickard JP, Leahy T, Crossett B, Druart X, de Graaf SP (2018). Cryopreservation and egg yolk medium alter the proteome of ram spermatozoa. J Proteomics.

[23] Casas I, Sancho S, Briz M (2009). Freezability prediction of boar ejaculates assessed by functional sperm parameters and sperm proteins. Theriogenology.

[24] Lamirande ED, Jiang H, Zini A, Kodama H, Gagnon C (1997). Reactive oxygen species and sperm physiology. Rev Reprod.

